# Synthesis of Oxadiazolyl, Pyrazolyl and Thiazolyl Derivatives of Thiophene-2-Carboxamide as Antimicrobial and Anti-HCV Agents

**DOI:** 10.2174/1874104501711010038

**Published:** 2017-04-28

**Authors:** Ola H. Rizk, Omaima G. Shaaban, Abeer E. Abdel Wahab

**Affiliations:** 1 Department of Pharmaceutical Chemistry, Faculty of Pharmacy, University of Alexandria, Alexandria, Egypt; 2 Department of Analytical and Pharmaceutical Chemistry, Faculty of Pharmacy & Drug Manufacturing, Pharos University, Alexandria, Egypt; 3 Genetic Engineering and Biotechnology Research Institute (GEBRI), Mubarak City for Scientific Research and Technology Application, Borg El-Arab, Alexandria, Egypt

**Keywords:** Synthesis, Thiophene-2-carboxamide, 1,3,4-Oxadiazole, Pyrazole, Antimicrobial activity, Antiviral activity

## Abstract

**Introduction:**

Three series of pyrazole, thiazole and 1,3,4-oxadiazole, derivatives were synthesized starting from 5-amino-4-(hydrazinocarbonyl)-3-methylthiophene-2-carboxamide **(2)**.

**Methods:**

All compounds were investigated for their preliminary antimicrobial activity. They were proved to exhibit remarkable antimicrobial activity against *Pseudomonas aeruginosa* with insignificant activity towards Gram positive bacterial strains and fungi.

**Results:**

*In-vitro* testing of the new compounds on hepatitis-C virus (HCV) replication in hepatocellular carcinoma cell line HepG2 infected with the virus utilizing the reverse transcription polymerase chain reaction technique (RT-PCR) generally showed inhibition of the replication of HCV RNA (–) strands at low concentration, while, eight compounds; **3a**, **6**, **7a**, **7b**, **9a**, **9b**, **10a** and **11b** proved to inhibit the replication of HCV RNA (+) and (–) strands at very low concentration range 0.08-0.36 μg/mL.

**Conclusion:**

Compounds **7b** and **11b** displayed the highest anti-HCV and antimicrobial activities in this study.

## INTRODUCTION

Hepatitis C virus (HCV) infection is a great global health issue with recent total population infection estimates of 1-3% [1, 2]. Egypt has the highest prevalence of Hepatitis C in the world, ranging from 6% to more than 40% among different regions [3]. HCV is believed to act as a carcinogen due to prevalence of hepatocellular carcinoma in patients infected with chronic active hepatitis. HCV causes chronic hepatic inflammation resulting in cellular disrupt which contribute to hepatocellular carcinoma [4]. Current therapies for HCV involve a combination of the nucleoside analog ribavirin and interferon-α [5]. This treatment regimen causes unfavorable side effects which often results in poor patient compliance [6]. Therefore, discovery of new anti HCV agents is urgently needed.

A literature survey revealed that thiophene nucleus was proved to be a core skeleton of several biologically active compounds [7-14]. Moreover, pyrazole, 1,3-thiazole and 1,3,4-oxadiazole moieties have been acknowledged to display various biological activities as antimicrobial [15-18], antitumor [19-22] in addition to antiviral [23-27] activities. Recently many research groups have paid attention for the synthesis of pyrazole, thiazole and oxadiazole due to their interesting pharmaceutical activities [28-31].

Encouraged by these findings, A new series of thiophene derivatives comprising the bioactive pyrazoles, thiazoles and 1,3,4-oxadiazoles at position 4 were synthesized and biologically evaluated.

## MATERIALS AND METHODS

### Chemistry

All reagents were purchased from commercial suppliers and purified by standard techniques. Melting points were determined in open glass capillaries using Thomas-Hoover melting point apparatus. Infrared spectra (IR) were recorded for KBr discs on Nicolet-800 FT-IR infrared spectrophotometer. NMR spectra were scanned on a Bruker 300 ultrashield spectrometer using tetramethylsilane (TMS) as internal standard and DMSO-d_6_ as solvent (chemical shifts are given in d/ppm). Splitting patterns were designated as follows: s: singlet, d: doublet, t: triplet, and m: multiplet. Mass spectra were run on a gas chromatograph/mass spectrometer Schimadzu GCMS-QP 2010 Plus (70 eV). Microanalyses were performed at the regional Center for Mycology and Biotechnology, El Azhar University and the found values were within ± 0.3% of the theoretical values. The reaction completion and purity of the compounds was followed by thin layer chromatography (TLC) on silica gel-precoated aluminium sheets (Type 60 GF254; Merck; Germany) and the spots were visualized by UV lamp at *λ* 254 nm for few seconds. The key intermediate **1** was prepared by Gütschow *et al.* [32]

### 5-Amino-4-(hydrazinocarbonyl)-3-Methylthiophene-2-Carboxamide (2)

A mixture of 1 (2.28 g, 10 mmol) and hydrazine hydrate 99% (4 mL, 80 mmol) in ethanol (10 mL) was heated under reflux for 24 h. The reaction mixture was cooled and the separated product was filtered, washed with ethanol, dried and crystallized from ethanol.

Yield: 62%, m.p.: 228-30^o^C. IR (KBr, cm^-1^): 3387, 3296, 3222, 3115 (NH_2_, NH), 1657 (C=O), 1624 (C=N), 1271, 1017 (C-S-C). ^1^H-NMR (DMSO-d_6_, ppm): 2.43 (s, 3H, CH_3_), 4.36 (s, 2H, NH_2_, D_2_O exchangeable), 7.61 (s, 2H, thiophene-C_5_-NH_2,_ D_2_O exchangeable), 8.07 (s, 2H, CONH_2_, D_2_O exchangeable), 8.90 (s, 1H, NH, D_2_O exchangeable). ^13^C-NMR (DMSO-d_6_, ppm): 14.76 (CH_3_), 105.68 (thiophene C_4_), 111.44 (thiophene C_2_), 139.78 (thiophene C_3_), 163.62, 165.26 (2 C=O), 165.40 (thiophene C_5_). Anal. Calcd. (%) for C_7_H_10_N_4_O_2_S (214.24): C, 39.24; H, 4.70; N, 26.15. Found: C, 39.52; H, 5.12; N, 26.37.

### 5-Amino-3-Methyl-4-[(3-Methyl-5-Substituted-1*H*-pyrazol-1-yl)Carbonyl]Thiophene-2-Carboxamide (3a,b)

A mixture of **2** (0.214 g, 1 mmol) and acetylacetone or benzoylacetone (1 mmol) in absolute EtOH (10 mL) containing 3 drops glacial acetic acid was heated under reflux for 12 h. The reaction mixture was allowed to attain room temperature and the separated product was filtered, dried and crystallized from DMF.

### 5-Amino-3-Methyl-4-[(3,5-Dimethyl-1*H*-Pyrazol-1-yl)Carbonyl]Thiophene-2-Carboxamide (3a)

Yield: 64%, m.p.: 146-8^o^C. IR (KBr, cm^-1^): 3377, 3258 (NH_2_), 1653 (C=O), 1587 (C=N), 1218, 1022 (C-S-C). ^1^H-NMR (DMSO-d_6_, ppm): 2.15 (s, 3H, pyrazole-C_3_-CH_3_), 2.59 (s, 3H, CH_3_), 2.85 (s, 3H, pyrazole-C_5_-CH_3_), 6.12 (s, 1H, pyrazole-C_4_-H), 7.93 (s, 2H, thiophene-C_5_-NH_2,_ D_2_O exchangeable), 8.24 (s, 2H, CONH_2_, D_2_O exchangeable). Anal. Calcd. (%) for C_12_H_14_N_4_O_2_S (278.33): C, 51.78; H, 5.07; N, 20.13. Found: C, 51.72; H, 5.47; N, 20.22.

### 5-Amino-3-Methyl-4-[(3-Methyl-5-Phenyl-1*H*-Pyrazol-1-yl) Carbonyl]Thiophene-2-Carboxamide (3b)

Yield: 68%, m.p.: 108-10^o^C. IR (KBr, cm^-1^): 3368, 3234 (NH_2_), 1652 (C=O), 1577 (C=N), 1218, 1021 (C-S-C). ^1^H-NMR (DMSO-d_6_, ppm): 2.25 (s, 3H, pyrazole-C_3_-CH_3_), 2.60(s, 3H, CH_3_), 6.42 (s, 1H, pyrazole-C_4_-H), 7.26 (t, 1H, J = 7.5 Hz, phenyl-C_4_-H), 7.38 (t, 2H, J = 7.5 Hz, phenyl-C_3&5_-H), 7.73 (d, J = 7.5 Hz, 2H, phenyl-C_2,6_-H),7.90 (s, 2H, thiophene-C_5_-NH_2,_ D_2_O exchangeable), 8.14 (s, 2H, CONH_2_, D_2_O exchangeable). Anal. Calcd. (%) for C_17_H_16_N_4_O_2_S (340.4): C, 59.98; H, 4.74; N, 16.46. Found: C, 59.97; H, 5.07; N, 16.58.

### Methyl 1-[(2-Amino-5-Carbamoyl-4-Methylthiophen-3-yl) Carbonyl]-5-Phenyl-4,5-Dihydro-1*H*-Pyrazole-3-Carboxylate (4)

A mixture of **2** (0.214 g, 1 mmol) and methyl pyruvate (1 mmol) in absolute EtOH (10 mL) containing 3 drops glacial acetic acid was refluxed for 10 h, and then cooled. The separated solid product was filtered, dried and crystallized from DMF.

Yield: 64%, m.p.: 114-6°C. IR (KBr, cm^-1^): 3413, 3314 (NH_2_), 1662 (C=O), 1582 (C=N), 1255, 1020 (C-S-C), 1175, 1036 (C-O-C). ^1^H-NMR (DMSO-d_6_, ppm): 2.62 (s, 3H, CH_3_), 3.87(s, 3H, COOCH_3_), 6.42 (s, 1H, pyrazole-C_4_-H), 7.19 (t, 1H, J = 7.5 Hz, phenyl-C_4_-H), 7.21 (t, 2H, J = 7.5 Hz, phenyl-C_3&5_-H), 7.63 (d, J = 7.5 Hz, 2H, phenyl-C_2,6_-H),7.82 (s, 2H, thiophene-C_5_-NH_2,_ D_2_O exchangeable), 8.04 (s, 2H, CONH_2_, D_2_O exchangeable). Anal. Calcd. (%) for C_18_H_16_N_4_O_4_S (384.41): C, 56.24; H, 4.20; N, 14.57. Found: C, 56.31; H, 4.52; N, 14.71.

### 5-Amino-4-[(3,5-Dioxopyrazolidin-1-yl) Carbonyl]-3-Methylthiophene-2-Carboxamide (5)

A mixture of **2** (0.214 g, 2.0 mmol) and diethylmalonate (3.27 g, 20 mmol, 3.1 mL) was heated under reflux for 4 h. then left to cool. The obtained precipitate was filtered, dried and crystallized from ethanol / water (4:1).

Yield: 70%, m.p.: 210-12^o^C. IR (KBr, cm^-1^): 3386, 3239 (NH_2_, NH), 1658, 1675(C=O), 1607 (C=N), 1249, 1025 (C-S-C). ^1^H-NMR (DMSO-d_6_, ppm): 1.86 (s, 2H, pyrazolidinyl-C_4_-H), 2.48 (s, 3H, CH_3_), 7.70 (s, 2H, thiophene-C_5_-NH_2,_ D_2_O exchangeable), 9.41(s, 2H, CONH_2_, D_2_O exchangeable), 9.71 (s, 1H, pyrazolidinyl-C_2_-NH, D_2_O exchangeable). ^13^C-NMR (DMSO-d_6_, ppm): 14.74 (CH_3_), 59.72 (pyrazole C_4_), 105.88 (thiophene C_4_), 110.24 (thiophene C_2_), 141.87 (thiophene C_3_), 163.50, 165.32, 165.55, 165.58 (4 C=O), 168.83 (thiophene C_5_). EI-MS *m/z*: 282 (M^+^ /1.03%), 57 (100%). Anal. Calcd. (%) for C_10_H_10_N_4_O_4_S (282.28): C, 42.55; H, 3.57; N, 19.85. Found: C, 42.63; H, 3.55; N, 20.08.

### 5-Amino-3-Methyl-4-[(3-Methyl-5-oxo-4,5-Dihydro-1*H*-Pyrazol-1-yl) Carbonyl]Thiophene-2-Carboxamide (6)

A mixture of **2** (0.214 g, 1 mmol) and ethylacetoacetate (1 mmol) in absolute EtOH (10 mL) containing 3 drops of glacial acetic acid was refluxed for 10 h, and the reaction mixture was left to attain room temperature. The separated product was filtered, dried and crystallized from ethanol.

Yield: 65%, m.p.: 208-10^o^C. IR (KBr, cm^-1^): 3378, 3272 (NH_2_), 1694, 1658 (C=O), 1588 (C=N), 1248, 1034 (C-S-C). ^1^H-NMR (DMSO-d_6_, ppm): 1.85 (s, 2H, pyrazol-C_4_-H), 2.22 (s, 3H, pyrazole-C_3_-CH_3_), 2.48 (s, 3H, CH_3_), 9.85 (s, 2H, thiophene-C_5_-NH_2,_ D_2_O exchangeable), 11.05 (s, 2H, CONH_2_, D_2_O exchangeable). Anal. Calcd. (%) for C_11_H_12_N_4_O_3_S (280.3): C, 47.13; H, 4.32; N, 19.99. Found: C, 47.32; H, 4.37; N, 20.14.

### 6-Methyl-2-Substituted-5-oxo-4,5-Dihydro-1*H*-Thieno [2,3-*e*][1, 2, 4]Triazepine-7-Carboxamide (7a-c)

Compounds **7a-c** were prepared by refluxing a mixture of **2** (0.214 g, 1 mmol) and the appropriate aromatic aldehyde (1 mmol) in absolute ethanol (20 mL) for 4 h. The reaction mixture was left to attain room temperature and the obtained products were filtered, dried and crystallized from the appropriate solvent.

### 6-Methyl-2-(4-Nitrophenyl)-5-oxo-4,5-Dihydro-1*H*-Thieno[2,3-*e*][1, 2, 4] Triazepine-7-Carboxamide (7a)

Yield: 74%, m.p.: 278-80^o^C (DMF). IR (KBr, cm^-1^): 3434, 3316, 3152 (NH_2_, NH), 1666 (C=O), 1632, 1579 (C=N), 1509, 1337 (NO_2_), 1267, 1025 (C-S-C). ^1^H-NMR (DMSO-d_6_, ppm): 2.61 (s, 3H, CH_3_), 7.78 (s, 2H, thiophene-C_5_-NH_2,_ D_2_O exchangeable), 7.95 (d, J = 8.9 Hz, 2H, phenyl-C_2,6_-H), 8.15 (s, 2H, CONH_2_, D_2_O exchangeable), 8.20 (s, 1H, NH_,_ D_2_O exchangeable), 8.26 (d, J = 8.9 Hz, 2H, phenyl-C_3,5_-H), 11.12 (s, 1H, NH_,_ D_2_O exchangeable). ^13^C-NMR (DMSO-d_6_, ppm): 14.75 (CH_3_), 106.05 (thienotriazepine C_3e_), 110.16 (thienotriazepine C_7_), 124.46 (4-nitrophenyl C_3,5_), 128.31 (4-nitrophenyl C_2,6_), 132.14 (4-nitrophenyl C_1_), 141.29 (thienotriazepine C_6_), 147.50 (thienotriazepine C_2_), 147.92 (4-nitrophenyl C_4_), 165.06, 165.34 (2 C=O), 170.65 (thienotriazepine C_2e_). Anal. Calcd. (%) for C_14_H_11_N_5_O_4_S (345.33): C, 48.69; H, 3.21; N, 20.28. Found: C, 48.82; H, 3.18; N, 20.45.

### 6-Methyl-2-(4-Methoxyphenyl)-5-Oxo-4,5-Dihydro-1*H*-Thieno[2,3-*e*][1, 2, 4] Triazepine-7-Carboxamide (7b)

Yield: 76%, m.p.: 160-2^o^C (ethanol). IR (KBr, cm^-1^): 3416, 3296, 3226 (NH_2_, NH), 1675 (C=O), 1603 (C=N), 1250, 1021 (C-S-C), 1167, 1117 (C-O-C). ^1^H-NMR (DMSO-d_6_, ppm): 2.59 (s, 3H, CH_3_), 3.79 (s, 3H, OCH_3_), 6.98 (d, J = 8.7 Hz, 2H, phenyl-C_2,6_-H), 7.65 (d, J = 8.7 Hz, 2H, phenyl-C_3,5_-H), 7.72 (s, 2H, thiophene-C_5_-NH_2,_ D_2_O exchangeable), 7.98 (s, 2H, CONH_2_, D_2_O exchangeable), 8.05 (s, 1H, NH_,_ D_2_O exchangeable), 11.16 (s, 1H, NH_,_ D_2_O exchangeable). ^13^C-NMR (DMSO-d_6_, ppm): 14.77 (CH_3_), 55.73 (OCH_3_), 105.60 (thienotriazepine C_3e_), 109.16 (thienotriazepine C_7_), 117.34 (4-methoxyphenyl C_3,5_), 122.14 (4-methoxyphenyl C_1_), 129.49 (4-methoxyphenyl C_2,6_), 138.05 (thienotriazepine C_6_), 146.04 (thienotriazepine C_2_), 158.77 (4-methoxyphenyl C_4_), 165.02, 165.59 (2 C=O), 170.91 (thienotriazepine C_2e_). EI-MS *m/z*: 329 (M^+^ /3.81%),330 (0.06), 139 (100%). Anal. Calcd. (%) for C_15_H_14_N_4_O_3_S (330.36): C, 54.53; H, 4.27; N, 16.96. Found: C, 54.72; H, 4.46; N, 17.13.

### 6-Methyl-2-(3,4-Dimethoxy)-5-Oxo-4,5-Dihydro-1*H*-Thieno [2,3-*e*][1, 2, 4] Triazepine-7-Carboxamide (7c)

Yield: 76%, m.p.: 180-2^o^C (ethanol/water, 4:1). IR (KBr, cm^-1^): 3383, 3279, 3214 (NH_2_, NH), 1658 (C=O), 1598 (C=N), 1269, 1024 (C-S-C), 1166, 1055 (C-O-C). ^1^H-NMR (DMSO-d_6_, ppm): 2.70 (s, 3H, CH_3_), 3.79,3.82 (2s, each 3H, 2OCH_3_), 7.0 (d, J = 8.35 Hz, 1H, phenyl-C_6_-H), 7.18 (d, J = 8.35 Hz, 1H, phenyl-C_5_-H), 7.33 (s, 1H, phenyl-C_2_-H), 7.71(s, 2H, thiophene-C_5_-NH_2,_ D_2_O exchangeable), 7.98 (s, 2H, CONH_2_, D_2_O exchangeable), 8.03 (s, 1H, NH_,_ D_2_O exchangeable), 11.03 (s, 1H, NH_,_ D_2_O exchangeable). Anal. Calcd. (%) for C_16_H_16_N_4_O_4_S (360.39): C, 53.32; H, 4.47; N, 15.55. Found: C, 53.41; H, 4.50; N, 15.71.

### 5-Amino-3-Methyl-4-{[2-(Substitute Dcarbamothioyl) Hydrazino]Carbonyl}Thiophene-2-Carboxamide (9a, b)

To a suspension of **2** (0.214 g, 1 mmol) in absolute ethanol (20 ml), the appropriate isothiocyanate (1.1 mmol) was drop wisely added over a period of 15 minutes. The reaction mixture was heated under reflux for 7 h. and left to attain room temperature. The obtained precipitate was filtered, dried and crystallized from DMF/H_2_O (5:2).

### 5-Amino-3-Methyl-4-{[2-(Phenylcarbamothioyl) Hydrazino] Carbonyl} Thiophene-2-Carboxamide (9a)

Yield: 72%, m.p.: 205-7^o^C. IR (KBr, cm^-1^): 3380, 3268, 3170 (NH_2_, NH), 1653 (C=O), 1588 (C=N), 1529, 1331, 1122, 932 (NCS), 1242, 1021 (C-S-C). ^1^H-NMR (DMSO-d_6_, ppm): 2.59 (s, 3H, CH_3_), 6.99 (t, 1H, J = 7.6 Hz, phenyl-C_4_-H), 7.34 (t, 2H, J = 7.6 Hz, phenyl-C_3&5_-H), 7.56 (d, J = 7.6 Hz, 2H, phenyl-C_2,6_-H), 7.74 (s, 1H, CSNH_,_ D_2_O exchangeable), 7.80 (s, 2H, thiophene-C_5_-NH_2,_ D_2_O exchangeable), 7.84 (s, 1H, CONHNH_,_ D_2_O exchangeable), 7.95 (s, 2H, CONH_2_, D_2_O exchangeable), 10.48 (s, 1H, CONHNH_,_ D_2_O exchangeable). Anal. Calcd. (%) for C_14_H_15_N_5_O_2_S_2_ (349.43): C, 48.12; H, 4.33; N, 20.04. Found: C, 48.33; H, 4.61; N, 20.21.

### 5-Amino-3-Methyl-4-({2-[(4-Methylphenyl) Carbamothioyl] Hydrazino} Carbonyl)Thiophene-2-Carboxamide (9b)

Yield: 76%, m.p.: 202-4^o^C. IR (KBr, cm^-1^): 3377, 3258, 3170 (NH_2_, NH), 1652 (C=O), 1587 (C=N), 1529, 1331, 1123, 931 (NCS), 1218, 1022 (C-S-C). ^1^H-NMR (DMSO-d_6_, ppm): 2.25 (s, 3H, phenyl-C_4_-CH_3_), 2.58 (s, 3H, CH_3_), 6.99 (t, 1H, J = 7.6 Hz, phenyl-C_4_-H), 7.34 (t, 2H, J = 7.6 Hz, phenyl-C_3&5_-H), 7.56 (d, J = 7.6 Hz, 2H, phenyl-C_2,6_-H), 7.63 (s, 1H, CSNH_,_ D_2_O exchangeable), 7.83 (s, 2H, thiophene-C_5_-NH_2,_ D_2_O exchangeable), 7.87 (s, 1H, CONHNH_,_ D_2_O exchangeable), 8.10 (s, 2H, CONH_2_, D_2_O exchangeable), 10.36 (s, 1H, CONHNH_,_ D_2_O exchangeable). EI-MS *m/z*: 363 (M^+^ /1.90%), 64 (100%). Anal. Calcd. (%) for C_15_H_17_N_5_O_2_S_2_ (363.46): C, 49.57; H, 4.71; N, 19.27. Found: C, 49.87; H, 4.97; N, 19.53.

### 5-Amino-4-{[(2*Z*)-2-(3,4-Disubstituted-1,3-Thiazol-2(3*H*)-Ylidene)Hydrazino]Carbonyl}-3-Methylthiophene-2-Carboxamide (10a-f)

A mixture of **9a **or** 9b** (1 mmol) and the appropriate phenacyl bromide (1 mmol) in absolute ethanol (20 ml) was heated under reflux for 20 h. Anhydrous sodium acetate (0.1 g) was then added and the reaction mixture was heated for further 30 minutes. The reaction mixture was cooled and poured onto ice-cold water. The separated product was filtered, washed with water and crystallized from the proper solvent.

### 5-Amino-4-{[(2*Z*)-2-(3,4-Diphenyl-1,3-Thiazol-2(3*H*)-Ylidene)Hydrazino]Carbonyl}-3-Methylthiophene-2-Carboxamide (10a)

Yield: 73%, m.p.: 195-7^o^C (ethanol). IR (KBr, cm^-1^): 3378, 3276, 3170 (NH_2_, NH), 1654 (C=O), 1583 (C=N), 1524, 1372, 1098, 868 (NCS), 1231, 1039 (C-S-C). ^1^H-NMR (DMSO-d_6_, ppm): 2.43 (s, 3H, CH_3_), 6.37 (s, 1H, thiazoline-C_3_-H), 6.95-7.53 (m, 10H, 2 phenyl-H), 7.76 (s, 2H, thiophene-C_5_-NH_2,_ D_2_O exchangeable), 7.96 (s, 2H, CONH_2_, D_2_O exchangeable), 10.53 (s, 1H, CONH_,_ D_2_O exchangeable). Anal. Calcd. (%) for C_22_H_19_N_5_O_2_S_2_ (449.55): C, 58.78; H, 4.26; N, 15.58. Found: C, 58.87; H, 4.30; N, 15.73.

### 5-Amino-4-({(2*Z*)-2-[4-(4-Bromophenyl)-3-Phenyl-1,3-Thiazol-2(3*H*)-Ylidene]Hydrazino}Carbonyl)-3-Methylthiophene-2-Carboxamide (10b)

Yield: 76%, m.p.: 142-4^o^C (ethanol/water, 3:2). IR (KBr, cm^-1^): 3404, 3286, 3169 (NH_2_, NH), 1658 (C=O), 1582 (C=N), 1523, 1325, 1097, 868 (NCS), 1229, 1009 (C-S-C). ^1^H-NMR (DMSO-d_6_, ppm): 2.44 (s, 3H, CH_3_), 6.45 (s, 1H, thiazoline-C_3_-H), 6.94 (d, J = 8.4 Hz, 2H, phenyl-C_2,6_-H), 7.03 (t, 1H, J = 8.4 Hz, phenyl-C_4_-H), 7.34 (t, 2H, J = 8.4 Hz, phenyl-C_3&5_-H), 7.45 (d, J = 8.4 Hz, 2H, 4-bromophenyl-C_2,6_-H), 7.63 (d, J = 8.4 Hz, 2H, 4-bromophenyl-C_2,6_-H), 7.78 (s, 2H, thiophene-C_5_-NH_2,_ D_2_O exchangeable), 7.97 (s, 2H, CONH_2_, D_2_O exchangeable), 10.53 (s, 1H, CONH_,_ D_2_O exchangeable). ^13^C-NMR (DMSO-d_6_, ppm): 14.71 (CH_3_), 105.62 (thiophene C_4_), 106.17 (thiazolidine C_3_), 108.69 (thiophene C_2_), 121.29 (phenyl C_2,6_), 122.76 (4-bromophenyl C_4_), 123.58 (phenyl C_4_), 129.58 (4-bromophenyl C_1_), 129.96 (4-bromophenyl C_2,6_), 130.11 (phenyl C_3,5_), 131.83 (4-bromophenyl C_3,5_), 138.73 (phenyl C_1_), 143.70 (thiophene C_3_), 150.63 (thiazolidine C_4_), 157.05 (thiazolidine C_1_), 162.37, 165.18 (2 C=O), 165.93 (thiophene C_5_). EI-MS *m/z*: 528 (M^+^ /2.25%), 125 (100%). Anal. Calcd. (%) for C_22_H_18_BrN_5_O_2_S_2_ (528.44): C, 50.00; H, 3.43; N, 13.25. Found: C, 50.13; H, 3.41; N, 13.37.

### 5-Amino-4-({(2*Z*)-2-[4-(4-Methoxyphenyl)-3-Phenyl-1,3-Thiazol-2(3*H*)-Ylidene]Hydrazino}Carbonyl)-3-Methylthiophene-2-Carboxamide (10c)

Yield: 72%, m.p.: 148-50^o^C (ethanol). IR (KBr, cm^-1^): 3404, 3286, 3169 (NH_2_, NH), 1658 (C=O), 1582 (C=N), 1523, 1325, 1097, 868 (NCS), 1229, 1009 (C-S-C) 1167, 1117 (C-O-C). ^1^H-NMR (DMSO-d_6_, ppm): 2.45 (s, 3H, CH_3_), 3.81 (s, 3H, OCH_3_), 6.25 (s, 1H, thiazoline-C_3_-H), 6.94-7.45 (m, 9H, phenyl & Ar-H), 7.75 (s, 2H, thiophene-C_5_-NH_2,_ D_2_O exchangeable), 7.89 (s, 2H, CONH_2_, D_2_O exchangeable), 10.46 (s, 1H, CONH_,_ D_2_O exchangeable). Anal. Calcd. (%) for C_23_H_21_N_5_O_3_S_2_ (479.57): C, 57.60; H, 4.41; N, 14.60. Found: C, 57.78; H, 4.46; N, 14.87.

### 5-Amino-4-({(2*Z*)-2-[3-(4-Methylphenyl)-4-Phenyl-1,3-Thiazol-2(3*H*)-Ylidene]Hydrazino}Carbonyl)-3-Methylthiophene-2-Carboxamide (10d)

Yield: 71%, m.p.: 203-205^o^C (ethanol). IR (KBr, cm^-1^): 3389, 3277, 3166 (NH_2_, NH), 1660 (C=O), 1588 (C=N), 1525, 1371, 1104, 872 (NCS), 1231, 1043 (C-S-C). ^1^H-NMR (DMSO-d_6_, ppm): 2.26 (s, 3H, phenyl CH_3_), 2.43 (s, 3H, CH_3_), 6.42 (s, 1H, thiazoline-C_3_-H), 6.91-7.55 (m, 9H, phenyl & Ar-H), 7.73 (s, 2H, thiophene-C_5_-NH_2,_ D_2_O exchangeable), 7.86 (s, 2H, CONH_2_, D_2_O exchangeable), 10.50 (s, 1H, CONH_,_ D_2_O exchangeable). Anal. Calcd. (%) for C_23_H_21_N_5_O_2_S_2_ (463.58): C, 59.59; H, 4.57; N, 15.11. Found: C, 59.72; H, 4.55; N, 15.32.

### 5-Amino-4-({(2*Z*)-2-[4-(4-Bromophenyl)-3-(4-Methylphenyl)-1,3-Thiazol-2(3*H*)-Ylidene]Hydrazino} Carbonyl)-3-Methylthiophene-2-Carboxamide (10e)

Yield: 73%, m.p.: 190-2^o^C (ethanol/water, 4:1). IR (KBr, cm^-1^): 3392, 3285, 3158 (NH_2_, NH), 1649 (C=O), 1586 (C=N), 1519, 1327, 1101, 869 (NCS), 1233, 1041 (C-S-C). ^1^H-NMR (DMSO-d_6_, ppm): 2.27 (s, 3H, phenyl CH_3_), 2.43 (s, 3H, CH_3_), 6.43 (s, 1H, thiazoline-C_3_-H), 6.83 (d, J = 8.2 Hz, 2H, 4-bromophenyl-C_2,6_-H), 7.14 (d, 2H, J = 8.2 Hz, 4-bromophenyl-C_3,5_-H), 7.44 (d, 2H, J = 8.4 Hz, 4-methylphenyl-C_3,5_-H), 7.63 (d, J = 8.4 Hz, 2H, 4-methylphenyl-C_2,6_-H), 7.77 (s, 2H, thiophene-C_5_-NH_2,_ D_2_O exchangeable), 7.96 (s, 2H, CONH_2_, D_2_O exchangeable), 10.49 (s, 1H, CONH_,_ D_2_O exchangeable). Anal. Calcd. (%) for C_23_H_20_BrN_5_O_2_S_2_ (542.47): C, 50.92; H, 3.72; N, 12.91. Found: C, 50.97; H, 3.74; N, 13.12.

### 5-Amino-4-({(2*Z*)-2-[4-(4-Methoxyphenyl)-3-(4-Methylphenyl)-1,3-Thiazol-2(3*H*)-Ylidene]Hydrazino} Carbonyl)-3 Methylthiophene-2-Carboxamide (10f)

Yield: 69%, m.p.: 184-6^o^C (ethanol). IR (KBr, cm^-1^): 3388, 3265, 3190 (NH_2_, NH), 1658 (C=O), 1590 (C=N), 1525, 1325, 1105, 888 (NCS), 1235, 1021 (C-S-C) 1170, 1112 (C-O-C). ^1^H-NMR (DMSO-d_6_, ppm): 2.24 (s, 3H, phenyl CH_3_), 2.47 (s, 3H, CH_3_), 3.90 (s, 3H, OCH_3_), 6.34 (s, 1H, thiazoline-C_3_-H), 6.97-7.35 (m, 8H, 2 Ar-H), 7.76 (s, 2H, thiophene-C_5_-NH_2,_ D_2_O exchangeable), 7.89 (s, 2H, CONH_2_, D_2_O exchangeable), 10.49 (s, 1H, CONH_,_ D_2_O exchangeable). Anal. Calcd. (%) for C_24_H_23_N_5_O_3_S_2_ (493.6): C, 58.40; H, 4.70; N, 14.19. Found: C, 58.53; H, 4.68; N, 14.32.

### 5-Amino-3-Methyl-4-{[(2*Z*)-2-(4-Oxo-3-Substituted-1,3-Thiazolidin-2-ylidene)Hydrazino]Carbonyl} Thiophene-2-Carboxamide(11a, b)

A mixture of **9a or b** (1 mmol) and ethyl bromoacetate (0.2 g, 0.13 mL, 1.1 mmol) in absolute ethanol (20 mL) was heated under reflux for 24 h. Anhydrous sodium acetate (0.1 g) was added and the reaction mixture was heated for further 30 minutes. The reaction mixture was cooled and poured onto ice-cold water. The separated product was filtered, washed with water and crystallized from dioxane.

### 5-Amino-3-Methyl-4-{[(2*Z*)-2-(4-Oxo-3-Phenyl-1,3-Thiazolidin-2-Ylidene)Hydrazino]Carbonyl}Thiophene-2-Carboxamide(11a)

Yield: 58%, m.p.: 194-6 ^o^C. IR (KBr, cm^-1^): 3392, 3255, 3170 (NH_2_, NH), 1658, 1640 (C=O), 1587 (C=N), 1535, 1326, 1105, 912 (NCS), 1227, 1059 (C-S-C). ^1^H-NMR (DMSO-d_6_, ppm): 2.60 (s, 3H, CH_3_), 4.78 (s, 2H, thiazolidine-C_3_-H), 7.01-7.63 (m, 5H, phenyl-H), 7.68 (s, 2H, thiophene-C_5_-NH_2,_ D_2_O exchangeable), 7.82 (s, 2H, CONH_2_, D_2_O exchangeable), 10.36 (s, 1H, CONH_,_ D_2_O exchangeable). Anal. Calcd. (%) for C_16_H_15_N_5_O_3_S_2_ (389.45): C, 49.34; H, 3.88; N, 17.98. Found: C, 49.68; H, 4.01; N, 18.21.

### 5-Amino-3-Methyl-4-({(2*Z*)-2-[3-(4-Methylphenyl)-4-Oxo-1,3-Thiazolidin-2-Ylidene]Hydrazino}Carbonyl) Thiophene-2-Carboxamide (11b)

Yield: 61%, m.p.: 202-4 ^o^C. IR (KBr, cm^-1^): 3386, 3245, 3175 (NH_2_, NH), 1670, 1642 (C=O), 1590 (C=N), 1537, 1326, 1109, 918 (NCS), 1225, 1075 (C-S-C). ^1^H-NMR (DMSO-d_6_, ppm): 2.26 (s, 3H, phenyl CH_3_), 2.58 (s, 3H, CH_3_) 4.88 (s, 2H, thiazolidine-C_3_-H), 7.12 (d, 2H, J = 8.4 Hz, 4-methylphenyl-C_3,5_-H), 7.51 (d, J = 8.4 Hz, 2H, 4-methylphenyl-C_2,6_-H),7.70 (s, 2H, thiophene-C_5_-NH_2,_ D_2_O exchangeable), 7.79 (s, 2H, CONH_2_, D_2_O exchangeable), 10.32 (s, 1H, CONH_,_ D_2_O exchangeable). Anal. Calcd. (%) for C_17_H_17_N_5_O_3_S_2_ (403.48): C, 50.61; H, 4.25; N, 17.36; Found: C, 50.94; H, 4.43; N, 17.63.

### 5-Amino-3-Methyl-4-[5-Substituted-1,3,4-Oxadiazol-2-yl] Thiophene-2-Carboxamide (12a,b)

A solution of **9a** or **9b** (1 mmol) in dry dioxane (10 ml) was treated with freshly prepared yellow HgO (0.26 g, 1.2 mmol). The reaction mixture was refluxed for 5 h. and filtered while hot to remove the precipitated black HgS. The filtrate was evaporated and the remaining residue was crystallized from dioxane.

### 5-Amino-3-Methyl-4-[5-(Phenylamino)-1,3,4-Oxadiazol-2-yl] Thiophene-2-Carboxamide (12a)

Yield: 70%, m.p.: 232-4^o^C. IR (KBr, cm^-1^): 3396, 3252, 3167 (NH_2_, NH), 1683 (C=O), 1571 (C=N), 1264, 1022 (C-S-C), 1124, 1073 (C-O-C). ^1^H-NMR (DMSO-d_6_, ppm): 2.56 (s, 3H, CH_3_), 7.12-7.48 (m, 5H, phenyl-H), 7.65 (s, 2H, thiophene-C_5_-NH_2,_ D_2_O exchangeable), 7.87 (s, 2H, CONH_2_, D_2_O exchangeable), 10.42 (s, 1H, NH_,_ D_2_O exchangeable). Anal. Calcd. (%) for C_14_H_13_N_5_O_2_S (315.35): C, 53.32; H, 4.16; N, 22.21. Found: C, 53.45; H, 4.14; N, 22.35.

### 5-Amino-3-Methyl-4-{5-[(4-Methylphenyl) Amino]-1,3,4-Oxadiazol-2-yl}Thiophene-2-Carboxamide(12b)

Yield: 72%, m.p.: 226-8^o^C. IR (KBr, cm^-1^): 3395, 3254, 3155 (NH_2_, NH), 1660 (C=O), 1586 (C=N), 1250, 1032 (C-S-C), 1118, 1072 (C-O-C). ^1^H-NMR (DMSO-d_6_, ppm): 2.26 (s, 3H, phenyl CH_3_), 2.59 (s, 3H, CH_3_), 7.15 (d, 2H, J = 8.4 Hz, 4-methylphenyl-C_2&6_-H), 7.46 (d, J = 8.4 Hz, 2H, 4-methylphenyl-C_3,5_-H), 7.82 (s, 2H, thiophene-C_5_-NH_2,_ D_2_O exchangeable), 7.98 (s, 2H, CONH_2_, D_2_O exchangeable), 10.35 (s, 1H, NH_,_ D_2_O exchangeable). Anal. Calcd. (%) for C_15_H_15_N_5_O_2_S (329.38): C, 54.70; H, 4.59; N, 21.26. Found: C, 54.84; H, 4.55; N, 21.39.

## BIOLOGICAL EVALUATION

### Anti-hepatitis C Virus Activity

#### Evaluation of Cytotoxicity Using Neutral Red Uptake Assay

Isolation of lymphocytes from whole human blood was done using gradient separation by Ficoll-PagueTM Plus (MP Biomedicals, France), _˜_10 × 10^4^ cells were seeded per well in 96 well plates and the plates were incubated in RPMI (Roswell Park Memorial Institute) media containing different concentrations of the tested compounds for 24, 48, 72 and 96 h. The fraction of viable lymphocyte cells was measured by the Neutral red assay [33]. The neutral red assay determines the accumulation of the neutral red dye in the lysosomes of viable cells [34]. Following exposure to different concentrations of tested compounds, cells were incubated for 3 h with neutral red dye (40μg/ml) dissolved in culture media RPMI. Cells were then washed with phosphate buffered saline (PBS) then 1 ml of elution medium (ethanol/glacial acetic acid /water 50/1/ 49%) was added followed by gentle shaking for 10 min so that complete dissolution was achieved. Aliquots of the resulting solutions were transferred to 96-well plates and absorbance at 490 nm was recorded using microliter plate reader spectrophotometer (Biotek, U.S.A). The assay was conducted three times. The IC_50_ (the concentration resulting in 50% death of cells) and EC_99_ (concentration of the compound that maintained 99% viability of cells) were calculated using GraphPad Prism (GraphPad Software Inc., San Diego, CA) [35] (Table **1**).

#### Cell Culture

In the last few years, a number of cell culture systems have been developed that support reliable and efficient progression of this virus. Among several human hepatocyte cell lines analyzed, the hepatocellular carcinoma HepG2 cell line was found to be the most susceptible to HCV infection [36]. HepG2 cells were washed twice with RPM11640 media supplemented with 200μM L-glutamine and 25μM HEPES buffer; N-[2-hydroxyethyl]piperazine-N`-[2-ethanesulphonic acid] (Lonza). The cells were suspended at 2 X 10^5^ cells/ mL in RPM1 culture media (RPM1 supplemented media, 10% fetal bovine serum (FBS); Gibco-BRL). The cells were left to adhere on the polystyrene 6-well plates for 4 h in an incubator (37°C, 5% CO_2_, 95% humidity). The cells were washed twice from debris and dead cells by using RPM1 supplemented media. HepG2 cell culture was prepared as discussed, then infected with HCV-infected serum in RPMI culture medium. Each of the tested compounds was added at the specified EC_99_ Table (**1**). Positive and negative control cultures were included. The positive strand and its replicating form (negative strand) were detected by reverse transcription-polymerase chain reaction (RT-PCR) using HCV specific primers to the 5'-untranslated region of the virus.

### Qualitative *In-vitro* Anti-HCV Screening

#### RNA Extraction and RT-PCR of HCV RNA

Total RNA was extracted from HepG2 HCV-infected cells as well as from HepG2 infected cells that are treated with the test compounds using the method described by El-Awady *et al.* [37]. Briefly, culture cells were mixed with 200µLof 4M guanidinium isothiocyanate containing 25 mM sodium citrate, 0.5% sarcosyl, 0.1 Mβ- mercaptoethanol, and 100 µL sodium acetate. The lysed cells were mixed with an equal volume mixture of water-saturated phenol, chloroform, and isoamyl alcohol. After vortexing of the sample, the mixture was centrifuged at 14 K rpm for 10 min at 4°C. The aqueous layer was collected and mixed with an equal volume of isopropanol. After incubation at –20^°^C overnight, RNA was precipitated by centrifugation at14 K rpm for 30 min at 4^o^C and the precipitated RNA was washed twice with 70% ethanol. The complimentary DNA (cDNA) and the first PCR reaction of the nested PCR detection system for the HCV RNA was performed in a 50 µL volume single-step reaction using the Ready-To-Go RT-PCR beads (Healthcare Life Sciences, USA), 10 µM from each of the RT downstream primer, PCR forward primer and reverse primer P2. The thermal cycling protocol was manipulated as follows: 30 min at 42^o^C for cDNA synthesis followed by 5 min at 95^o^C and 30 cycles of 1 min at 94°C, 1 min at 55^o^C and 1 min at 72^o^C. The nested PCR amplification was performed in 50 µL reaction mixture containing 0.2 mM from each dNTP, 10 µM from each of the reverse nested primer and the forward nested primer, two units of taq DNA polymerase (Promega, Madison, WI, USA), 10 µL from the RT-PCR reaction in a 1X buffer supplied by the Vendor. A fragment of 174bp length was identified in positive samples.

### Antimicrobial Screening

#### Inhibition-zone Measurements

All the synthesized compounds were evaluated by the agar cup diffusion technique [38-40] using a 1 mg/mL solution in DMSO. The test organisms were *Staphylococcus aureus* (DSM 1104) and *Bacillus subtilis* (ATCC 6633) as Gram-positive bacteria; *Escherichia coli* (ATCC 11775) and *Pseudomonas aeruginosa* (ATCC 10145) as Gram-negative bacteria. *Candida albicans* (DSM 70014) was also used as a representative for fungi. Each 100 mL of sterile molten agar (at 45^o^C) received 1 mL of 6 h-broth culture and then the seeded agar was poured into sterile Petri dishes. Cups (8 mm in diameter) were cut in the agar. Each cup received 0.1 mL of the 1 mg/mL solution of the test compounds. The plates were then incubated at 37°C for 24 h or, in case of *C. albicans*, for 48 h. A control using DMSO without the test compound was included for each organism. Ampicillin was used as standard antibacterial, while clotrimazole was used as antifungal reference. The resulting inhibition zones are recorded in Table (**2**).

#### Minimal Inhibitory Concentration (MIC) Measurement

The minimal inhibitory concentrations (MIC) of the most active compounds were measured using the twofold serial broth dilution method [39, 40]. The test organisms were grown in their suitable broth: 24 h for bacteria and 48 h for fungi at 37^o^C. Twofold serial dilutions of solutions of the test compounds were prepared using 200, 100, 50, 25, and 12.5 µg/mL. The tubes were then inoculated with the test organisms; each 5 mL received 0.1 mL of the above inoculum and were incubated at 37°C for 48 h. Then, the tubes were observed for the presence or absence of microbial growth. The MIC values of the prepared compounds are listed in Table **3**.

#### Minimal Bactericidal Concentration (MBC) Measurement

MIC tests were always extended to measure the MBC as follows: A loop-full from the tube not showing visible growth (MIC) was spread over a quarter of Müller-Hinton agar plate. After 18 h of incubation, the plates were examined for growth. Again, the tube containing the lowest concentration of the test compound that failed to yield growth on subculture plates was judged to contain the MBC of that compound for the respective test organism (Table **3**).

## RESULTS AND DISCUSSION

### Chemistry

Synthesis of the intermediate and target compounds are illustrated in schemes (**1** and **2**).

In scheme (**1**), the starting ester (**1**) [32] was synthesized by heating a mixture of acetoacetamide, ethyl cyanoacetate and sulfur in the presence of morpholine. Heating the ester **1** with hydrazine hydrate in ethanol gave rise to the hydrazide **2**. Condensation of the hydrazide **2** with acetyl acetone or benzoylacetone in ethanol containing glacial acetic acid afforded the corresponding 3,5-dimethylpyrazole **3a** and 3-methyl-5-phenylpyrazole **3b**. The pyrazole derivative **4** was prepared by heating the acid hydrazide **2** with ethyl pyruvate in ethanol containing glacial acetic acid. The 3,5-dioxopyrazolidine derivative **5** was successfully achieved by heating **2** with diethylmalonate. On the other hand, refluxing of **2** with ethyl acetoacetate in ethanol containing glacial acetic acid gave the required 5-oxo-4,5-dihydro**-**1*H*-pyrazole derivative **6**. The chemical structure of compounds **3a**, **b**, **4**, **5** and **6** were verified by analytical and spectral data.

Surprisingly, heating of the hydrazide **2** with aromatic aldehydes in ethanol containing glacial acetic acid yielded the 1,2,4 triazepine derivatives **7a-c** instead of the expected hydrazones **8a-c**. ^1^H-NMR spectra of these compounds lacked the D_2_O exchangeable singlets characteristic for NH_2_ and N=CH.

**Scheme (2):**
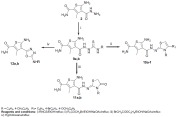
Synthetic pathways for compounds (**9-12**).

In scheme **2**, the *N*-substituted thiocarbamoyl hydrazino derivatives **9a, b **was prepared by refluxing the acid hydrazide **2** with aryl isothiocyanate in absolute ethanol. Cyclocondensation of **9a**, **b** with the selected phenacyl bromide or ethyl bromoacetate in ethanol containing anhydrous sodium acetate produced the respective thiazoline derivatives **10a-f**, and thiazolidinone derivatives **11a, b** respectively. Cyclodesulphurization of **9a, b** using mercuric oxide in dry dioxane furnished the target 1,3,4-oxadiazole derivatives **12a, b** in good yields.

The structure of the new compounds was elucidated by spectral analysis in addition to microanalysis.

## BIOLOGICAL EVALUATION

### Anti-hepatitis C virus activity

#### Evaluation of Cytotoxicity

All synthesized compounds were investigated for their effect on the proliferative activity of HepG2 cancer cell line, and the IC_50_ and EC_99_ in µg/ mL (Table **1**) were determined for each test compound using GraphPad Prism.

### Qualitative *In-vitro* Anti-HCV Screening

All synthesized compounds were evaluated for their *in-vitro* anti-HCV activity utilizing the hepatocellular carcinoma HepG2 cell line infected with hepatitis-C virus. Detection of viral (+) and/or (–) RNA strands was performed using qualitative RT-PCR. Detection of the (–) strand HCV-RNA using RT-PCR has been recently reported as a very important tool for understanding the life cycle of HCV. It is the most reliable biological marker for the diagnosis of HCV and monitoring the viral response to antiviral therapy [37].

According to these facts, simultaneous detection of the (+) and/or (–) HCV-RNA strands in HepG2 hepatoma cells infected with HCV was performed. Inhibition of viral replication was detected by amplification of viral RNA segments using the RT-PCR technique, both in the cultivated infected cells alone (as a positive control) and in presence of the specified doses of the test compounds at optimal temperature. An active compound is capable of inhibiting viral replication inside the HepG2 cells, as evidenced by the disappearance of the (+) and/or (–) strands viral RNA-amplified products detected by the RT-PCR (compared to positive control).

Inspection of (Figs. (**1**-**4**) showed that all the synthesized compounds inhibited the replication of HCV RNA (–) strands at the EC_99_. In addition, eight compounds **3a**, **6**, **7a**, **7b**, **9a**, **9b, 10a** and **11b** were proved to inhibit replication of both (+) and (–) strands at very low concentration range (EC_99_ = 0.08- 0.36 µg/mL) as shown in Table **1**. The obtained data revealed that the pyrazolyl derivatives **3a** and **6** inhibited the replication of both (+) and (–) strands of HCV RNA at 0.10 and 0.27 µg/mL. Conversion of the acid hydrazide **2** into the dihydro-1*H*-thieno[2,3-*e*] [1, 2, 4] triazepine-7-carboxamide**s 7a** and **7b** enhanced the activity against both (+) and (–) strands. Transformation of the hydrazide **2** into the *N*-substituted thiocarbamoylhydrazino derivatives **9a**, **b** yielded compounds that are able to inhibit replication of both (+) and (–) strands at EC_99_ = 0.16 and 0.36 µg/mL. Finally, cyclization of **2** into the 1,3-thiazolidin-2-ylidene)hydrazino]carbonyl}thiophene-2-carboxamide derivatives **10a** and **11b** yielded compounds active against both (+) and (–) strands.

### Antimicrobial Screening

The new compounds were investigated for their *in vitro* antibacterial activity against Gram-positive bacteria as* Staphylococcus aureus* and *Bacillus subtilis,* and Gram-negative bacteria as *Escherichia coli* and *Pseudomonas aeruginosa* . They were also studied for their *in vitro* antifungal potential against *Candida albicans*. The inhibition zones were measured utilizing the cup-diffusion technique [38, 39] and compounds showing reasonable inhibition zones (A ≥13 mm) were further evaluated to determine their minimal inhibitory concentration (MIC) and minimum bacterial concentration (MBC) applying the twofold serial dilution method [39, 40]. Ampicillin was used as antibacterial reference while clotrimazole was used as antifungal standard. Dimethylsulfoxide (DMSO) was used as blank and showed no antimicrobial activity.

Results from (Tables **2** and **3**) revealed that twenty two compounds showed promising inhibitory effects on the growth of the tested Gram-positive and Gram-negative microorganisms with high activity against *P. aeruginosa*. Regarding the antibacterial activity of the active compounds against *S. aureus*, compounds **6 **and** 10a **(MIC = 25 µg/mL) exhibited moderate activity, whereas the other compounds displayed weak activity. Concerning the activity against *B. subtilis*, compounds **5**, **7c** and **10a** (MIC = 25 µg/mL) displayed half the activity of ampicillin while compounds **3a**, **6**, **10f** and **11a** were four times less active than ampicillin against the same organism. While, *P. aeruginosa* was proved to be the most sensitive microorganism to most of the tested compounds. Compounds **3b**, **5, 7b, 10b, 11a** and **11b** (MIC = 25 µg /mL) showed double the activity of ampicillin, whereas compounds **4**, **7a**, **9a**, **9b, 10c, 10d**, **10e**, **11b**, **12a** and **12b** were equipotent to ampicillin. Furthermore, compounds **7c**, **9a**, **10d, 10f, 11a, 11b** and **12b** (MIC = 25 µg /mL) showed nearly half the activity of ampicillin against *E. coli*, meanwhile other compounds showed moderate to mild activity. On the other hand, only three compounds **3b**, **4** and **10b** produced moderate growth inhibition (MIC = 25 µg /mL) against *C. Albicans*. According to the MIC and MBC limits derived from the latest National Committee on Clinical Laboratory Standards (NCCLS), it can be decided whether the compound is bactericidal or bacteriostatic. Consequently, data from (Table **3**) revealed that only compounds **10d**, **11a** and **12a** were bactericidal against *P. aeruginosa* while the other compounds were bacteriostatic against the test organisms.

The activity of the test compounds could be tentatively correlated to the structural variations and modification. The pyrazolyl derivatives **3b** and **5**. showed potent activity against *P. aeroginosa* (MIC = 25 µg/mL), while compound **5** showed good activity against *B. subtilis* (MIC = 25 µg/mL). Whereas, the triazepine derivatives **7a** and **7b** exhibited substantial activity against *P. aeroginosa* (MIC = 50 and 25µg/mL respectively). Meanwhile, the triazepine derivative **7c** displayed moderate activity against *B. Subtilis* and *E. Coli* (MIC = 25 µg/mL). Cyclization of *N*-substituted thiocarbamoyl hydrazino derivatives **9a, b** into the corresponding thiazoline derivatives **10a-f**, and thiazolidinone derivatives **11a,b** didn't significantly affect the antimicrobial potential except for compounds **10b, 10d, 10f, 11a** and **11b** that showed activity against Gram-negative microorganisms while compound **10a** showed activity against Gram-positive microorganisms.

## CONCLUSION

In the present study, twenty new 5-amino-4-substituted-3-methylthiophene-2-carboxamide derivatives were prepared and their chemical structures were confirmed by spectral analyses in addition to elemental analyses. All compounds were investigated for their preliminary *in-vitro* effect on the replication of hepatitis-C virus (HCV) in HepG2 hepatocellular carcinoma cell line infected with the virus using the reverse transcription polymerase chain reaction technique. All compounds were found to inhibit the replication of HCV RNA (–) strands at the EC_99_, meanwhile, eight compounds (**3a**, **6**, **7a**, **7b**, **9a**, **9b, 10a** and **11b**) were proved to inhibit replication of both (+) and (–) strands at very low concentration range (EC_99_ = 0.08- 0.36 µg/mL).

Moreover, all compounds exhibited remarkable activity against the tested microorganisms with high activity against the gram-negative organism *P. aeruginosa*. Compounds **3b**, **5, 7b, 10b** and **11a** and **11b** emerged with the highest potential against *P. aeruginosa* as they displayed double the activity of ampicillin against this organism. Collectively, the triazepine derivative **7b** and the thiazolidinone derivative **11b** could be considered as the most active compounds in this study with an interesting dual anti-HCV and antimicrobial profile.

Finally, the obtained anti-HCV and antimicrobial data suggest that the 3-methylthiophene-2-carboxamide might be a potential skeleton for further development of more active and selective compounds with antihepatitis-C and/ or antimicrobial activities.

## DECLARATION/CONFLICT OF INTEREST

The authors declared no conflicts of interest. The authors alone are responsible for the content and writing of the paper.

## Figures and Tables

**Fig. (1):**
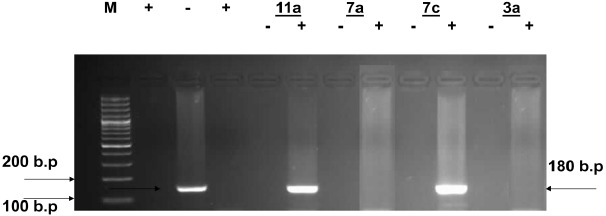
Agarose gel electrophoresis showing PCR amplified product of HCV RNA (+) and (-) strands in presence of compounds **11a, 7a, 7c** **3a**. Lane 1: molecular weight marker (M) 100bp ladder, lanes 2 and 4 = positive control and lane 3 = negative control. Lanes 5, 7, 9 and 11 show the effect of compounds **11a, 7a, 7c** and **3a** on HCV RNA (-) strands and lanes 6, 8, 10 and 12 show the effect of compounds **11a, 7a, 7c** and **3a** on HCV RNA (+) strands isolated from HepG2 cells at the EC99 in µg/mL after 3 days of treatment.

**Fig. (2):**
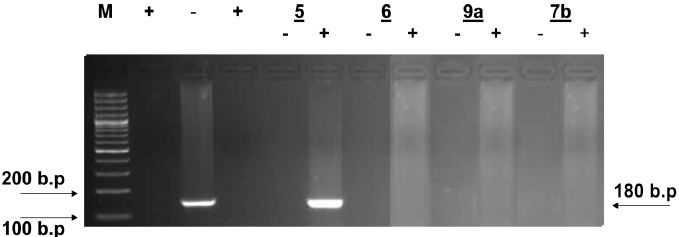
Agarose gel electrophoresis showing PCR amplified product of HCV RNA (+) and (-) strands in presence of compounds **5, 6, 9a** and **7b**. Lane 1: molecular weight marker (M) 100bp ladder, lanes 2 and 4 = positive control and lane 3 = negative control. Lanes 5, 7, 9 and 11 show the effect of compounds **5, 6, 9a** and **7b** on HCV RNA (-) strands and lanes 6, 8, 10 and 12 show the effect of compounds **5, 6, 9a** and **7b** on HCV RNA (+) strands isolated from HepG2 cells at the EC99 in µg/mL after 3 days of treatment.

**Fig. (3):**
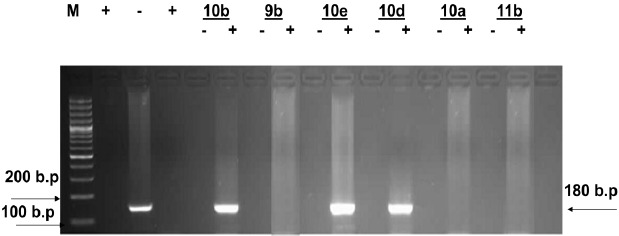
Agarose gel electrophoresis showing PCR amplified product of HCV RNA (+) and (-) strands in the presence of compounds **10b, 9b, 10e, 10d, 10a** and **11b**. Lane 1: molecular weight marker (M) 100bp ladder, lanes 2 and 4 = positive control and lane 3 = negative control. Lanes 5, 7, 9, 11, 13 and 15 show the effect of compounds **10b, 9b, 10e, 10d, 10a** and **11b** on HCV RNA (-) strands and lanes 6, 8, 10, 12, 14 and 16 show the effect of compounds **10b, 9b, 10e, 10d, 10a** and **11b** on HCV RNA (+) strands isolated from HepG2 cells at the EC99 in µg/mL after 3 days of treatment.

**Fig. (4):**
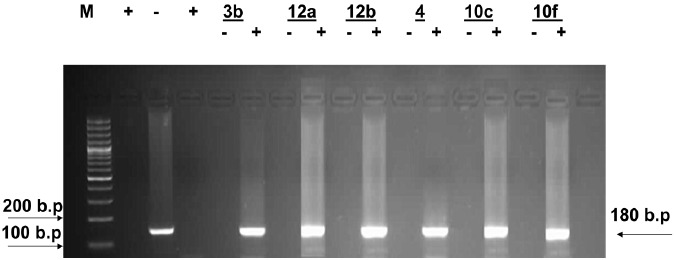
Agarose gel electrophoresis showing PCR amplified product of HCV RNA (+) and (-) strands in the presence of compounds **3b, 12a, 12b, 4, 10c** and **10f**. Lane 1: molecular weight marker (M) 100bp ladder, lanes 2 and 4 = positive control and lane 3 = negative control. Lanes 5, 7, 9, 11, 13 and 15 show the effect of compounds **3b, 12a, 12b, 4, 10c** and **10f** on HCV RNA (-) strands and lanes 6, 8, 10, 12, 14 and 16 show the effect of compounds **3b, 12a, 12b, 4, 10c** and **10f** on HCV RNA (+) strands isolated from HepG2 cells at the EC99 in µg/mL after 3 days of treatment.

**Scheme (1):**
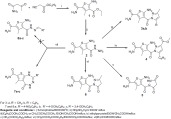
Synthetic pathways for compounds (**1-7**).

**Table 1:** Preliminary anti-HCV activity of the synthesized compounds **3-12** on HepG 2.

**Compound No.**	**IC_50_^a)^** **(µg/mL)**	**EC_99_^b)^** **(µg/mL)**	**Anti-HCV Activity^c)^**
**3a**	5.64	0.10	positive
**3b**	6.60	0.12	negative
**4**	5.50	0.11	negative
**5**	8.77	0.17	negative
**6**	14.33	0.27	positive
**7a**	6.50	0.12	positive
**7b**	4.37	0.08	positive
**7c**	10.40	0.20	negative
**9a**	8.46	0.16	positive
**9b**	18.16	0.36	positive
**10a**	6.80	0.13	positive
**10b**	7.80	0.14	negative
**10c**	4.20	0.08	negative
**10d**	5.75	0.11	negative
**10e**	4.23	0.08	negative
**10f**	9.45	0.18	negative
**11a**	5.30	0.10	negative
**11b**	7.04	0.13	positive
**12a**	8.10	0.15	negative
**12b**	12.16	0.23	negative

^a)^ IC_50_: concentration of the compound that caused 50% death of cells. ^b)^ EC_99_: concentration of the compound that maintained 90-99% viability of cells. ^c)^ Ability to inhibit the replication of both HCV RNA (+) and (–) strands.

**Table 2:** The inhibition zones (IZ) in mm diameter of the synthesized compounds **3-12**.

***Cpd No.***	***S. aureus***	***B. subtilis***	***P. aeroginosa***	***E. coli***	***C. albicans***
**3a**	16	14	12	17	18
**3b**	17	14	12	15	17
**4**	16	16	14	12	18
**5**	18	16	12	18	14
**6**	14	18	17	16	16
**7a**	18	14	15	15	14
**7b**	18	14	16	15	14
**7c**	14	18	12	14	16
**9a**	12	12	18	16	18
**9b**	14	16	18	16	12
**10a**	12	18	14	16	14
**10b**	14	14	16	12	14
**10c**	14	14	18	16	14
**10d**	18	16	12	14	14
**10e**	18	18	12	16	14
**10f**	16	14	15	12	18
**11a**	18	12	14	14	16
**11b**	16	14	12	16	14
**12a**	18	14	12	14	12
**12b**	14	16	15	14	14
**A^a^**	9	12	7	10	-
**C^b^**	-^c^	-	-	-	10

**^a^ A**: Ampicillin trihydrate (standard broad spectrum antibiotic). **^b^C**: Clotrimazole (standard broad spectrum antifungal agent). **^c^** (–): Totally inactive.

**Table 3:** Minimum inhibitory concentrations (MIC) and minimum bactericidal concentrations (MBC) of the synthesized compounds **3-12** in µg/mL.

***Cpd No.***	***S. aureus***		***B.subtilis***		***P.aeruginosa***		***E.coli***		***C.albicans***	
	**MIC**	**MBC**	**MIC**	**MBC**	**MIC**	**MBC**	**MIC**	**MBC**	**MIC**	**MBC**
**3a**	100	100	50	100	100	100	100	200	50	100
**3b**	50	100	100	200	25	50	50	50	25	50
**4**	100	100	100	100	50	100	50	100	25	25
**5**	100	100	25	50	25	50	50	100	50	50
**6**	25	50	50	50	100	200	50	100	100	100
**7a**	100	200	100	100	50	100	100	100	50	100
**7b**	50	50	100	100	25	50	50	100	100	100
**7c**	100	200	25	50	100	100	25	50	100	100
**9a**	100	100	100	200	50	100	25	50	50	100
**9b**	50	50	100	100	50	100	50	100	50	50
**10a**	25	50	25	100	100	100	50	100	100	100
**10b**	50	100	100	200	25	50	100	100	25	50
**10c**	100	100	100	200	50	100	100	200	100	100
**10d**	100	100	100	100	50	50	25	50	50	100
**10e**	50	100	100	200	50	100	100	100	50	100
**10f**	100	200	50	100	100	100	25	50	100	200
**11a**	100	200	50	50	25	25	25	50	50	100
**11b**	100	100	100	100	25	100	25	25	50	100
**12a**	50	100	100	100	50	50	100	200	50	100
**12b**	100	100	100	100	50	100	25	25	50	100
**A^a^**	5	-	12.5	-	50	-	10	-	-	-
**C^b^**	-**^c^**	-	-	-			-	-	5	-

**^a^ A**: Ampicillin trihydrate (standard broad spectrum antibiotic). **^b^C**: Clotrimazole (standard broad spectrum antifungal agent). **^c^** (–): Totally inactive (MIC > 200 µg/mL).
